# Cell therapy medicinal product regulatory framework in Europe and its application for MSC-based therapy development

**DOI:** 10.3389/fimmu.2012.00253

**Published:** 2012-08-14

**Authors:** Janis Ancans

**Affiliations:** Laboratory of Bioanalytical and Biodosimetry Methods, Faculty of Biology, University of LatviaRiga, Latvia

**Keywords:** advanced therapy medicinal product, cell therapy medicinal product, mesenchymal stem/progenitor cell, Committee for Advanced Therapies, Hospital Exemption, national competent authority

## Abstract

Advanced therapy medicinal products (ATMPs), including cell therapy products, form a new class of medicines in the European Union. Since the ATMPs are at the forefront of scientific innovation in medicine, specific regulatory framework has been developed for these medicines and implemented from 2009. The Committee for Advanced Therapies (CAT) has been established at the European Medicines Agency (EMA) for centralized classification, certification and evaluation procedures, and other ATMP-related tasks. Guidance documents, initiatives, and interaction platforms are available to make the new framework more accessible for small- and medium-sized enterprises, academia, hospitals, and foundations. Good understanding of the centralized and national components of the regulatory system is required to plan product development. It is in the best interests of the cell therapy developers to utilize the resources provided starting with the pre-clinical stage. Whilst there have been no mesenchymal stem cell (MSC)-based medicine authorizations in the EU, three MSC products have received marketing approval in other regions since 2011. The information provided on the regulatory requirements, procedures, and initiatives is aimed at facilitating MSC-based medicinal product development and authorization in the EU.

## INTRODUCTION

The scientific progress and advances in the biotechnology sector have led to the development of therapies which are based on the use of living cells, recombinant genetic material, and *in vitro* engineered tissue. A number of cell therapy and tissue engineered products have been introduced into the national markets of several Member States during the last decade. Due to the novelty, complexity, and technical specificity of such products, specially tailored and harmonized rules were necessary to ensure free movement of those products within the EU. Consequently, the Regulation (EC) N° 1394/2007 on advanced therapy medicinal products (ATMPs) was drafted and came into force on December 30, 2008. The Regulation laid down specific rules concerning centralized authorization, supervision, and pharmacovigilance of the ATMPs (Committee for Advanced Therapies and CAT Scientific Secretariat, 2010).

The term “advanced therapy medicinal product” covers the following medicinal products for human use: somatic cell therapy medicinal products (CTMPs), gene therapy medicinal products, and tissue engineered products. Combined ATMPs incorporate one or more medical devices as an integral part of the product. The scope of this article is primarily CTMP. For cells to be classified as medicinal products they have to fulfill at least one of the following conditions: the cells have been subject to substantial manipulation and/or these cells are not intended for use for the same essential function (the term “non-homologous use” is also used). By “substantial manipulation” it is understood that the biological characteristics, functions, or properties relevant for the therapeutic effect have been altered. Taking into account the methodological complexity of the cell therapy products, and in order to reduce the possible interpretations, it has been defined that certain manipulations with the cells and tissues are not to be considered as substantial. These include (tissue) cutting, grinding, shaping, centrifugation, soaking in antibiotic or antimicrobial solutions, sterilization, irradiation, cell separation, concentration or purification, filtering, freezing, cryopreservation, and vitrification (all listed in the Annex I of the Regulation (EC) N° 1394/2007). From the scientific or clinical perspective it can be argued that cell irradiation, for instance, can have a substantial effect on the biological characteristics and physiological functions that may be relevant also for the intended therapeutic application. Since interpretations by product developer and the regulator may differ, the exact legal definition for somatic CTMP is provided in **Table 1**.

**Table 1:** Glossary of key terms for advanced therapy developers in the EU.

Advisory procedures available for ATMP development process:
(a) *Classification* of product by the EMA: optional incentive for applicants, fast procedure, applied preferably during the early development stage;
(b) EMA *Innovation Task Force (ITF) briefing meeting*: early dialog with product developers with confidential and legally non-binding advice; the NCAs of some Member States may also provide similar “introductory” meetings with regulatory experts;
(c) *Certification* of quality and non-clinical data by the EMA: preferably applied before the clinical development stage, but the presence of clinical data does not preclude this procedure;
(d) *Scientific Advice* procedure: can address any quality, non-clinical, and clinical question at any time point of the product development (post-marketing advice is also available); provided by the EMA, but similar procedures can be offered in some countries by the national competent authority (NCA).
Advanced therapy medicinal product (ATMP)
Any of the following medicinal products for human use:
(a) gene therapy medicinal product,
(b) somatic cell therapy medicinal product,
(c) tissue engineered product.
Depending on the product characteristics, ATMP can be placed on the market in accordance with centralized marketing authorization procedure according to Regulation (EC) N° 1394/2007 or, if applicable to product, via Hospital Exemption clause in individual Member State.
Somatic cell therapy medicinal product (CTMP)
ATMP which has the following characteristics:
(a) contains or consists of somatic cells or tissues that have been subject to substantial manipulation so that the biological characteristics, physiological functions, or structural properties relevant for the intended clinical use have been altered; or of cells or tissues that are not intended for the same essential function(s) in the recipient and the donor;
(b) is presented as having properties for, or is used in or administered to human beings with a view to treating, preventing or diagnosing a disease through the action of its cells or tissues.
Centralized marketing authorization (MA)
The centralized MA procedure is required for certain categories of medicines in Europe, including the ATMPs. This procedure results in a single marketing authorization that is valid in all EU countries, as well as in Iceland, Liechtenstein, and Norway. The European pharmaceutical regulatory framework is applied, in particular the requirements of the Directive 2001/83/EC and the Regulation (EC) N° 726/2004, also the Regulation (EC) N° 1394/2007 with regard to the ATMPs. The EMA is responsible for the centralized procedure for medicines and the Committee for Advanced Therapies (CAT) evaluates ATMP submissions.
In order to address serious unmet medical needs of patients, it may be possible to obtain MA on the basis of less complete data than normally. Besides a standardized marketing authorization some medicinal product indications may present a case for a conditional approval. *Conditional marketing authorization* is subject to specific post-marketing obligations as set out in the Regulation (EC) N° 507/2006. However, such an authorization is not supposed to remain conditional indefinitely. Once the missing data are provided, it should be possible to replace it with a marketing authorization which is not conditional.
Hospital Exemption (HE)
Centralized MA is not required for such ATMPs which are prepared on a non-routine basis according to specific quality standards and are used within the same Member State in a hospital under the exclusive professional responsibility of a medical practitioner in order to comply with an individual medical prescription for a custom-made product for an individual patient.
Manufacturing of such ATMP products is authorized by the NCA of the Member State. The Member States have to ensure compliance with the adequate quality standards, as well as the traceability and pharmacovigilance requirements. The HE clause is implemented through the national legislative acts and there are differences among the HE regulations of individual Member States.
Transitional period
Advanced therapy medicinal products, excluding tissue engineered products, which were legally on the markets of the Member States in accordance with the or the EU legislation on December 30, 2008, had to comply with the Regulation (EC) N° 1394/2007 no later than by December 30, 2011. Tissue engineered products have to comply with the regulation no later than by December 30, 2012.

The requirements for the cell therapy product marketing authorization dossier are prescribed in the Directive 2001/83/EC. It has to be verified whether the text of the directive includes the amendments introduced until at least 2011. A link to the consolidated version is provided in the references. Thus, cell therapy requirements were introduced in 2010 by the Directive 2009/120/EC amending the Directive 2001/83/EC. In brief, the particulars and documents of a cell therapy dossier are presented as five modules: Module 1 provides the European Community specific administrative data; Module 2 provides the quality, non-clinical, and clinical summaries, Module 3 provides chemical, pharmaceutical, and biological information, Module 4 provides the non-clinical reports, and Module 5 provides the clinical study reports. The Regulation (EC) N° 1394/2007 also introduced amendments to the Directive 2001/83/EC, for instance Article 28 added provisions for the Hospital Exemption (HE, Article 3.7. of the consolidated Directive 2001/83/EC).

## OVERVIEW OF THE EUROPEAN REGULATORY FRAMEWORK

Directives and regulations are the two types of the EU legislative acts that form the regulatory framework for all medicines, including cell-based products. This legal framework provides the basis for centralized and national competencies. The directives set the general requirements for the Member States which implement these requirements by adopting national legislative acts. Certain variability of these implementation measures exists amongst the Member States. The regulations have to be implemented directly and uniformly, without the national legislative acts. The Regulation (EC) N° 1394/2007 provides the legal basis for a centralized authorization procedure of the ATMPs – it involves a single scientific evaluation of the quality, safety, and efficacy of the product carried out to the highest possible standard by the European Medicines Agency (EMA). National (NCA) and centralized [EMA/Committee for Advanced Therapies (CAT)] competences at the different stages of cell therapy product development are summarized in **Table 2**.

**Table 2:** Centralized and national components of the regulatory framework for ATMP development in the EU.

Type of activity	Legislation	NCA	EMA/CAT
Cell and tissue donation, procurement, processing	National	Inspection, authorization	n/a
Pre-clinical development	National	GLP inspection, consultation	Certification procedure (optional)
Clinical development	National	GCP, GMP inspections, authorization	n/a
ATMP classification	EU	Consultation	Procedure/opinion
ATMP certification	EU	n/a	Procedure/opinion/certificate
ATMP evaluation	EU	n/a	Procedure/opinion
Transition period	EU	Consultation	ATMP evaluation
Hospital exemption	National	Consultation, GMP inspection, production license	n/a

*GLP, good laboratory practice; GCP, good clinical practice; GMP, good manufacturing practice.*

Centralized procedures are provided by the EMA which is an interface for the cooperation and coordination of the activities of all 27 Member States with respect to the medicinal products. It is responsible for coordinating the existing scientific resources for the evaluation, supervision, and pharmacovigilance of the medicinal products. The EMA has seven committees including the CAT as well as a number of working parties which are expert groups with a specific scope and mandate. In addition to the evaluation of product marketing applications, the EMA mandates include scientific and procedure advice, the Innovation Task Force (ITF) meetings with product developers, coordination of the inspections of the Member States (GMP, GCP, GLP), and other. The mandates of the EMA do not cover the following ATMP development-related issues: pre-clinical development, clinical trial authorization, products legally on the market during the transitional period, HE authorization, functions of the ethics committee (but the EMA has the expertise to evaluate ethical issues), pricing and reimbursement of medicinal products. These issues are regulated at the Member State level by the national competent authority (NCA). Some states have one regulatory office whilst others have several NCAs that cover different regulatory tasks. The respective EU directives set the scene for the national regulatory frameworks. The Directive 2004/23/EC (with the implementing Directives 2006/17/EC and 2006/86/EC) defines the quality and safety standards for the donation, procurement, testing, processing, preservation, storage, and distribution of human tissues and cells. In the case of blood cells or blood components for the ATMP manufacture, the requirements of the Directive 2002/98/EC apply. For ATMPs that contain human cells or tissues, Directive 2004/23/EC and 2002/98/EC derived national provisions will apply as far as donation, procurement and testing are concerned. The requirements of these directives do not apply to research projects, their scope is only the tissues and cells intended for human use. Clinical trials with ATMPs should be conducted in accordance with the overarching principles and the requirements laid down in the Directive 2001/20/EC on approximation of the national laws, regulations and the administrative provisions for the implementation of good clinical practice in the conduct of clinical trials on medicinal products for human use. Compared with the regulatory system in the US, the EMA is not “FDA of Europe” since the FDA has a direct mandate to regulate the above mentioned issues and perform other tasks, including research. The current developments indicate that the EMA is likely to acquire more mandates in the future which will reduce the historical fragmentation of the European regulatory framework for medicinal product development.

The CAT provides a centralized cell therapy product evaluation procedure. The CAT formulates a draft opinion on the quality, safety, and efficacy of a product for the final approval by the Committee for the Medicinal Products for Human Use (CHMP). The EU marketing authorization which is based on a centralized evaluation procedure takes 210 days excluding clock-stops, it is defined in the Regulation (EC) N° 726/2004. The evaluation is done by two independent (Reporter and Co-Reporter) assessor teams with the CAT and the CHMP representatives. The fee for marketing authorization is reduced by 50% if the applicant is a hospital or a small- or medium-sized enterprise and can prove that there is particular public health interest in the ATMP concerned. This is prescribed in the Article 19 of the Regulation (EC) N° 2007/1394. However, even if the marketing authorization for a cell therapy product is granted, the Regulation does not interfere with the decisions of the Member State on whether to allow the use of any specific type of human cells such as embryonic stem cells or xenogeneic cells. It does not affect the application of the national legislation prohibiting or restricting the sale, supply or use of medicinal products containing, consisting of or derived from particular cells. Several cell therapy products were legally on the Member State markets before December 30, 2008. Such products have been granted a transition period defined in the Article 29 of the Regulation (EC) N° 1394/2007 during which products have to comply with the Regulation. The transition period for CTMPs has already expired at the end of 2011, and for tissue engineered products the transition period expires in 2012.

The evaluation-related tasks of the CAT include the classification of the advanced therapy products and certification of their pre-clinical data quality. Product developers have access to the classification procedure in order to determine whether a given product based on cells, genes or tissues meets the scientific criteria which define it as an ATMP. It is an incentive but not a legal requirement for the applicants and an opinion is delivered within 60 days after the receipt of the request. More than 50 classifications have been completed by 2012 and non-confidential summaries are available on the EMA website. ATMP classification procedure does not determine whether product dossier will be evaluated by the centralized procedure in or it can be submitted for the HE in Member State. However, the CAT classification procedure opinion is not legally binding for the NCAs in case product is submitted for the HE. Finally, certification of the quality and non-clinical data is a new and unique procedure available only for the medicinal products of advanced therapy classification and it is based on the Regulation (EC) N° 668/2009. Micro businesses and SMEs developing an ATMP can submit all the relevant quality and where available non-clinical data to the EMA for scientific evaluation by the CAT. It is a 90-day procedure and in the case of a favorable CAT opinion the EMA will issue a corresponding certificate. The certification is not legally binding but it will facilitate the development and improve the clinical trial and marketing authorization applications based on the same data. Only one cell therapy certification has been completed since the launching of this procedure. This is possibly due to the optional nature of the procedure and the interpretation that the resulting opinion is not legally binding for the EMA. It is also possible that SMEs developing cell therapy products may not be yet fully aware of this procedure and the related potential benefits. It has to be emphasized that a positive outcome of the certification procedure indicates that the regulatory agency has evaluated and recognized the quality of non-clinical data. Certification of the data quality also minimizes the possibility of major objections at the marketing application evaluation stage and may serve as an incentive for investments in the development of cell therapy.

Upon request of the Executive Director of the EMA or the European Commission the CAT provides advice and scientific support to drafting documents related to the fulfillment of the objectives of the ATMP regulation. On request of the European Commission the committee provides scientific expertise and advice for initiatives related to the development of innovative medicines and therapies which require the expertise in advanced therapy-related scientific areas. The activities proposed in the CAT Work programme 2010–2015 may be of interest for the ATMP stakeholders. The document is available at the EMA website and link is provided in the references. Considering the potential of the ATMPs and due to the lack of product progress to the market, the CAT has adopted proactive approach in providing the guidance tools and ensuring a dialog with the relevant parties. A number of program activities are targeted at the needs of the ATMP developers and the stakeholders are welcome to communicate their opinion via the EMA website. The CAT Work programme for the period of 2010–2015 is aimed at providing positive long term impact on the advanced therapy sector in Europe.

## CONSIDERATIONS FOR DEVELOPMENT OF MSC-BASED MEDICINES

The mesenchymal stem cells (MSCs) have produced beneficial effects in a wide range of pre-clinical development disease models, even though there are as yet no adequate explanations for many of the effects observed (Prockop and Oh, 2012). During the last decade the MSC-based therapy clinical trials have been conducted for at least a dozen of different medical conditions (Wang et al., 2012). The results of clinical studies have led to the conclusion that MSC applications have been safe and feasible. However, the efficacy often could not be convincingly demonstrated as the therapies advanced along with the clinical development. This is also illustrated by the absence of MSC-based products in the European market. Only a few MSC-based cell therapy products have been approved in other markets worldwide. South Korea is leading with two MSC products registered and the first authorization granted in 2011. It might be linked to the procedure of a conditional marketing approval in the regulatory framework of South Korea that allows commercial sale in certain instances whilst pivotal trials are underway. There is also a procedure of conditional marketing authorization in the EU prescribed by the Regulation (EC) N° 507/2006 but certain differences exist in the regulatory systems. With the approval from the Korean FDA in January 2012, Cartistem has become the world’s first allogenic, off-the-shelf MSC-based product. The product contains the umbilical cord blood (UCB)-derived MSCs and it is indicated for the treatment of traumatic and degenerative osteoarthritis. In 2011 the Korean company FCB PharmiCell received Korean FDA approval for commercial sale of HeartiCellgram indicated for post-acute myocardial infarction treatment. It is autologous bone marrow-derived MSC therapy product. The company provides 50–90 million cells (depending on the weight of the patient) which are administered by infusion into the coronary arteries. The regulatory approval for HeartiCellgram was granted after 6 years of clinical trials. The company has announced that the patients displayed a 6% improvement in the left ventricular ejection fraction 6 months after one dose of HeartiCellgram. However, the company has not published the results in a peer-reviewed journal (Wohn, 2012). It seems that a similar regulatory decision has been adopted for Osiris Therapeutics Inc. product Prochymal which consists of allogenic MSCs. The company was granted an authorization for the treatment of acute graft-vs-host disease (GvHD) in children under Health Canada’s Notice of Compliance with conditions (NOC/c) in May 2012. This is an authorization to market on condition that the manufacturer undertakes additional studies to verify the clinical benefit. Such a regulatory pathway provides access to treatments for unmet medical conditions and has demonstrated the benefits outweigh the risks in the clinical trials. Overall this may represent a regulatory trend to consider the evaluation procedures that could address medical needs more efficiently. Adaptive licensing, e.g., conditional approvals, would be based on stepwise learning in circumstances of acknowledged uncertainty, with iterative phases of data gathering and regulatory re-evaluation (Eichler et al., 2012). Adaptive licensing requires a different approach from the standardized dichotomous unapproved/approved product paradigm.

Better understanding of the regulatory framework should improve the development strategy and create opportunities for MSC-based therapy product authorization also in Europe. From the regulatory perspective all MSC-based products in the EU will be classified as ATMPs unless the developer claims that the MSCs have been obtained without *in vitro* culture step. According to the CAT opinion, the cell culture process corresponds to a “substantial manipulation” and the derived cells qualify as an active substance of a medicinal product. The MSCs containing medicinal product can be classified as cell therapy or tissue engineered product depending on the intended use and the claimed mode of action. The addition of recombinant proteins, chemicals and biologically active molecules *in vitro* to the MSCs will not change the regulatory status of the product. Introduction of gene expression vector into MSCs does not change the ATMP status but will result in reclassification from the cell to gene therapy product, since the product which may fall within the definition of the CTMP and the gene therapy medicinal product should be considered as a latter (as defined in Part IV of Annex I to Directive 2001/83/EC). The studies that report MSC clinical application often present the development strategy decisions which could be re-evaluated in case the overall aim was to develop a cell therapy product. It is often reported that unmodified primary MSC cultures have been used. Correspondingly, pre-clinical screening has been absent since there was only one active substance candidate. The rationale of the use of a particular MSC culture should be evaluated if mesenchymal cell clinical application does not require the same essential function as in the tissue of origin. Instead, the MSC trials often aim to facilitate the tissue regeneration or to achieve the immunomodulatory effect. Pre-clinical screening and selection is an integral part of conventional medicinal product development and there is no reason to assume that it should not be introduced also for the cell therapy developments. With the technology available there are several strategies that might be considered in order to introduce the MSC therapy candidate selection step. For instance, modification of a primary MSC population with small chemical compounds and subsequent screening for expression or secretome profiles could be considered to improve the study design and the efficacy (Ranganath et al., 2012). Genetic modification to express factor(s) that are expected to mediate or enhance the therapeutic effect and characterization of transfected clones also represents rational development (Olson et al., 2012). The comparison of subpopulations or primary cultures of different origin will increase the number of candidates for the screening and has been applied for the cell-based medicinal product development (Li et al., 2012). These examples illustrate the feasibility of the pre-clinical screening step also for the cell-based therapies. Whilst the screening will increase the costs and may not be attractive or necessary for the academic research activities, it may present a cost efficient improvement for the discovery of new MSC therapy candidates. It may provide benefits also from novel intellectual property acquisition perspective.

The MSC product developers are advised to start with the EMA scientific guidelines that provide a detailed description of the quality, safety, efficacy, and pharmacovigilance issues for CTMPs. These guidance documents include the “Guideline on human cell-based medicinal products,” “Guideline on the safety and efficacy follow-up – risk management of advanced therapy medicinal products,” “Guideline on strategies to identify and mitigate risks for first-in-human clinical trials with investigational medicinal products,” “Guideline on the quality, preclinical and clinical aspects of medicinal products containing genetically modified cells,” and “Reflection paper on stem cell-based medicinal products.” “Guideline on the risk-based approach according to Annex I, part IV of Directive 2001/83/EC applied to Advanced Therapy Medicinal Products” is an important ATMP guidance document which has been published for public discussion and will be finalized by the end of 2012 (the e-links for all guidance documents are provided in the references). The above-mentioned guidance documents should be examined in detail but it is neither the scope of these documents nor is it technically feasible to describe the whole variety of cell therapy products. Instead, the risk analysis may cover the entire development process and an adequate risk-based approach strategy has to be applied. According to the Directive 2001/83/EC, due to the specific nature of the ATMPs, the application of a risk-based approach is encouraged to determine the extent of quality, the non-clinical and clinical data to be included in the marketing authorization application. The risk analysis methodology followed, the nature of the identified risks and the implications of the risk-based approach for the development and evaluation program has to be discussed and the risk analysis has to be described in the product application. For instance, if conventional pharmacological and toxicological tests may not be considered as appropriate for a cell therapy product, the relevant biological parameters such as the cell viability, biodistribution, ectopic growth, and the expression patterns can be investigated. It is acknowledged that relevant animal models for cell therapy product and indication might not exist. Immunocompromised animals may have a limited value, the structural and functional dissimilarities between the animal and human target organs/tissues may not produce relevant data. It would be reasonable to assume that the marketing authorization and certification application evaluation will be carried out in accordance with the risk-based approach until a certain number of products reach the market.

The whole range of assistance procedures and initiatives available from the EMA and the NCAs should be considered (summary in **Table 1**). The product classification procedure is highly recommended at an early stage of development. This will confirm the legal framework and the relevant guidance documents can be applied for the development. Certification of non-clinical and quality data should be considered since a positive outcome would facilitate and streamline the product development for authorization. Since there are no MSC products authorized in the EU, certification by the regulatory agency may encourage funding agencies and the potential investors. Application for EMA ITF briefing meeting definitely should be considered. The ITF provides a multidisciplinary group that contains scientific, regulatory, and legal competences. It is a forum for an early dialog with the applicants. The ITF briefing meetings are meant to complement other regulatory procedures, such as the classification, certification, and scientific advice (SA). The ITF meetings are free of charge; the application forms and information about the procedure are available at the EMA website. The SA procedure provides considerably more detailed information to the applicant and can be used at any stage of the MSC product development. The procedure helps the applicant to make sure that appropriate tests and studies are performed. Consequently no major objections are likely to be raised during the evaluation of the marketing authorization application. Such major objections could significantly delay the marketing of a product and may result in a refusal of the authorization. Adherence to the SA recommendations can substantially increase the probability of a positive marketing outcome (Regnstrom et al., 2010). Within the current initiative the EMA provides SA to the SMEs for a fee reduced by 90%, and with a 65% reduction for other applicants that develop the ATMPs. Several NCAs also have the capacity and the expertise to provide similar advice but this has to be confirmed with the particular agency. The protocol assistance procedure should be considered for orphan or rare disease products, i.e., a condition affecting no more than 5 in 10,000 people in the EU. This procedure is available at the EMA and presents a special form of SA. As a result the “orphan designation” may be applied for medicinal products that meet the criteria and the incentives for “orphan designation” include the fee reduction and 10 years of market exclusivity once authorized. Products developed for ultra rare diseases may qualify for a marketing authorization under exceptional circumstances which requires a less complete data set. The following guidance document has been published for further information: “Guideline on procedures for the granting of a marketing authorization under exceptional circumstances, pursuant to Article 14 (8) of Regulation (EC) N° 726/2004.”

Alternatively, an MSC therapy product may be considered for national authorization procedure under the HE clause in a particular Member State. In order to qualify for HE authorization, a product has to be prepared on a non-routine basis according to specific quality standards, and has to be used within the same Member State in a hospital under the exclusive professional responsibility of a medical practitioner (legal definition provided in **Table 1**). The HE regulatory instrument could be in a way perceived as adaptive licensing at the national level for advanced therapy medicinal products. It has to be acknowledged that inherent and unavoidable autologous cell material differences can make certain cell therapy applications more similar to the development of medical technology than to classical medicinal product development process with standard clinical trials (Webster et al., 2011). This does not mean that autologous cell products by definition would qualify for HE approval since regulatory decision is not based on the origin of cell material. However, autologous products combined with complex medical procedures are more likely to qualify for the HE due to the inherent product characteristics. In either case the HE is still a very new regulatory procedure which, if considered, should be discussed in advance with the experts of an NCA. The implementation of the HE clause has been accomplished in the majority of EU states by 2012, but the terms and conditions of the authorization vary and each Member State decides on the implementation tools. For instance, a recent publication illustrates the differences between France and the UK regarding the HE authorizations and also provides information on several other ATMP development-related issues that are regulated differently at the national level (Mahalatchimy et al., 2012). This reference should be examined in case the application for the HE authorization is planned in the UK or France. For instance, the UK agency provides options for either the HE authorization or the “Specials” exemption status according to Article 5.1. of Directive 2001/83/EC. The website of the UK NCA – Medicines and Healthcare products Regulatory Agency (MHRA) – provides user friendly information on the ATMP-related questions and relevant flow-charts in the section “How we regulate advanced therapy medicinal products.” Information on the HE clause in Germany is available on the website of Paul-Ehrlich-Institute (PEI) which is the German NCA. The PEI Innovation Office website includes summaries in English and flow-chart on how the ATMPs are regulated in Germany (links are provided in references). German ATMP framework is reviewed and analyzed in detail in a recent publication (Buchholz et al., 2012).

## CONCLUSION

Regulatory centralization has been introduced for cell therapy product marketing in the EU since 2009 but the remaining national procedures can be quite heterogeneous. Still, general provisions in the national legal acts are based on the EU directives and therefore will be common for all Member States. The EMA and NCA guidance documents, initiatives, and interaction platforms are available to make the regulatory framework more understandable and accessible for investigators both in the public and private sectors. Good understanding of centralized and national components of the framework will form an essential part of a product development plan and initial shortfalls will be difficult to compensate at the marketing authorization application stage. It is in the best interests of the investigators and investors to communicate with the NCAs and the EMA already during early phase of MSC product formulation. Resulting strategy improvements may facilitate MSC-based medicine development and authorization in the European Union.

## Conflict of Interest Statement

Author of this article is a member of EMA Committee for Advanced Therapies (CAT) and a core member of Cell based Products Working Party (CPWP). The views and opinions expressed in this article are those of the author and do not necessarily represent the views and opinions of EMA.

## Appendix

### LEGISLATIVE ACTS

Regulation (EC) N° 726/2004 of the European Parliament and of the Council of 31 March 2004 laying down Community procedures for the authorisation and supervision of medicinal products for human and veterinary use and establishing a European Medicines Agency. Available at: http://eur-lex.europa.eu/LexUriServ/LexUriServ.do?uri=OJ:L:2004:136:0001:0033:en:PDF

Regulation (EC) N° 507/2006 on the conditional marketing authorisation for medicinal products for human use falling within the scope of Regulation (EC) No 726/2004 of the European Parliament and of the Council. Available at: http://eur-lex.europa.eu/LexUriServ/LexUriServ.do?uri=OJ:L:2006:092:0006:0009:EN:PDF

Regulation (EC) N° 1394/2007 of the European Parliament and of the Council of 13 November 2007 on advanced therapy medicinal products and amending Directive 2001/83/EC and Regulation (EC) N° 726/2004. Available at: http://eur-lex.europa.eu/LexUriServ/LexUriServ.do?uri=OJ:L:2007:324:0121:0137:en:PDF

Commission Regulation (EC) N° 668/2009 of 24 July 2009 implementing Regulation (EC) No 1394/2007 of the European Parliament and of the Council with regard to the evaluation and certification of quality and non-clinical data relating to advanced therapy medicinal products developed by micro, small and medium-sized enterprises. Available at: http://eur-lex.europa.eu/LexUriServ/LexUriServ.do?uri=OJ:L:2009:194:0007:0010:EN:PDF

Directive 2001/20/EC of the European Parliament and of the Council of 4 April 2001 on the approximation of the laws, regulations and administrative provisions of the Member States relating to the implementation of good clinical practice in the conduct of clinical trials on medicinal products for human use. Available at: http://eur-lex.europa.eu/LexUriServ/LexUriServ.do?uri=OJ:L:2001:121:0034:0044:EN:PDF

Directive 2001/83/EC of the European Parliament and of the Council of 6 November 2001 on the Community code relating to medicinal products for human use (consolidated). Available at: http://ec.europa.eu/health/files/eudralex/vol-1/dir_2001_83_cons2009/2001_83_cons2009_en.pdf

Directive 2002/98/EC of the European Parliament and of the Council of 27 January 2003 setting standards of quality and safety for the collection, testing, processing, storage and distribution of human blood and blood components and amending Directive 2001/83/EC. Available at: http://eur-lex.europa.eu/LexUriServ/LexUriServ.do?uri=OJ:L:2003:033:0030:0040:EN:PDF

Directive 2004/23/EC of the European Parliament and of the Council of 31 March 2004 on setting standards of quality and safety for the donation, procurement, testing, processing, preservation, storage and distribution of human tissues and cells. Available at: http://eur-lex.europa.eu/LexUriServ/LexUriServ.do?uri=OJ:L:2004:102:0048:0058:en:PDF

Commission Directive 2009/120/EC of 14 September 2009 amending Directive 2001/83/EC of the European Parliament and of the Council on the Community code relating to medicinal products for human use as regards advanced therapy medicinal products. Available at: http://eur-lex.europa.eu/LexUriServ/LexUriServ.do?uri=OJ:L:2009:242:0003:0012:EN:PDF

### GUIDANCE DOCUMENTS

Guideline on procedures for the granting of a marketing authorisation under exceptional circumstances, pursuant to Article 14 (8) of Regulation (EC) N° 726/2004 (EMEA/357981/2005). Available at: http://www.ema.europa.eu/docs/en_GB/document_library/Regulatory_and_procedural_guideline/2009/10/WC500004883.pdf

Guideline on human cell-based medicinal products (EMEA/CHMP/410869/2006). Available at:http://www.emea.europa.eu/docs/en_GB/document_library/Scientific_guideline/2009/09/WC500003894.pdf

Guideline on strategies to identify and mitigate risks for first-in-human clinical trials with investigational medicinal products (EMEA/CHMP/SWP/28367/07). Available at: http://www.emea.europa.eu/docs/en_GB/document_library/Scientific_guideline/2009/09/WC500002988.pdf

Guideline on the safety and efficacy follow-up – risk management of advanced therapy medicinal products (EMEA/149995/2008). Available at: http://www.emea.europa.eu/docs/en_GB/document_library/Regulatory_and_procedural_guideline/2009/10/WC500006326.pdf

Guideline on the minimum quality and non-clinical data for certification of advanced therapy medicinal products (EMA/CAT/486831/2008). Available at: http://www.ema.europa.eu/docs/en_GB/document_library/Scientific_guideline/2010/01/WC500070031.pdf

Guideline on the quality, preclinical and clinical aspects of medicinal products containing genetically modified cells (EMA/CAT/GTWP/671639/2008). Available at: http://www.ema.europa.eu/docs/en_GB/document_library/Scientific_guideline/2012/05/WC500126836.pdf

Reflection paper on stem cell-based medicinal products (EMA/CAT/571134/2009). Available at: http://www.ema.europa.eu/docs/en_GB/document_library/Scientific_guideline/2011/02/WC500101692.pdf

Committee for Advanced Therapies (CAT) Work Programme 2010 – 2015 (EMA/CAT/235374/2010). Available at: http://www.ema.europa.eu/docs/en_GB/document_library/Work_programme/2010/11/WC500099029.pdf

Draft guideline on the risk-based approach according to Annex I, part IV of Directive 2001/83/EC applied to Advanced Therapy Medicinal Products (EMA/CAT/CPWP/686637/2011). Available at: http://www.ema.europa.eu/docs/en_GB/document_library/Scientific_guideline/2012/01/WC500120989.pdf

How we regulate advanced therapy medicinal products: MHRA. Available at: http://www.mhra.gov.uk/Howweregulate/Advancedtherapymedicinalproducts/Aboutadvancedtherapymedicinalproducts/index.htm

Informal advice for the development of Advanced Therapy Medicinal Products (ATMP): The Innovation Office at the Paul-Ehrlich-Institut. Available at: http://www.pei.de/cln_227/nn_2309950/EN/infos-en/pu-en/innovation-office/innovation-office-node.html?__nnn=true
